# Tissue-Engineered Regeneration of Completely Transected Spinal Cord Using Induced Neural Stem Cells and Gelatin-Electrospun Poly (Lactide-Co-Glycolide)/Polyethylene Glycol Scaffolds

**DOI:** 10.1371/journal.pone.0117709

**Published:** 2015-03-24

**Authors:** Chang Liu, Yong Huang, Mao Pang, Yang Yang, Shangfu Li, Linshan Liu, Tao Shu, Wei Zhou, Xuan Wang, Limin Rong, Bin Liu

**Affiliations:** 1 Department of Spine Surgery, The Third Affiliated Hospital, Sun Yat-Sen University, Guangzhou 510630, People’s Republic of China; 2 Department of Breast & Thyroid Surgery, The Third Affiliated Hospital, Sun Yat-Sen University, Guangzhou 510630, People’s Republic of China; 3 Department of Biochemistry, University of California, Los Angeles, California 90095–1569, United States of America; EFS, FRANCE

## Abstract

Tissue engineering has brought new possibilities for the treatment of spinal cord injury. Two important components for tissue engineering of the spinal cord include a suitable cell source and scaffold. In our study, we investigated induced mouse embryonic fibroblasts (MEFs) directly reprogrammed into neural stem cells (iNSCs), as a cell source. Three-dimensional (3D) electrospun poly (lactide-co-glycolide)/polyethylene glycol (PLGA-PEG) nanofiber scaffolds were used for iNSCs adhesion and growth. Cell growth, survival and proliferation on the scaffolds were investigated. Scanning electron microcopy (SEM) and nuclei staining were used to assess cell growth on the scaffolds. Scaffolds with iNSCs were then transplanted into transected rat spinal cords. Two or 8 weeks following transplantation, immunofluorescence was performed to determine iNSC survival and differentiation within the scaffolds. Functional recovery was assessed using the Basso, Beattie, Bresnahan (BBB) Scale. Results indicated that iNSCs showed similar morphological features with wild-type neural stem cells (wt-NSCs), and expressed a variety of neural stem cell marker genes. Furthermore, iNSCs were shown to survive, with the ability to self-renew and undergo neural differentiation into neurons and glial cells within the 3D scaffolds *in vivo*. The iNSC-seeded scaffolds restored the continuity of the spinal cord and reduced cavity formation. Additionally, iNSC-seeded scaffolds contributed to functional recovery of the spinal cord. Therefore, PLGA-PEG scaffolds seeded with iNSCs may serve as promising supporting transplants for repairing spinal cord injury (SCI).

## Introduction

Neural functional recovery after spinal cord injury (SCI) has, for many years, been a difficult problem to overcome [1,2,3]. Although many recent studies have shown that spinal cords have a weak ability for regeneration after SCI, neural functional recovery in adult mammals remains hard to achieve [4,5]. For the past few decades, research has been focused on an effective method to treat spinal cord injury, including gene therapy [6], cell transplantation [5,7] and drug treatment [8]. As an emerging cross discipline, tissue engineering provides a new direction and possibility for the treatment of SCI [9,10,11]. Using tissue engineering technology, suitable cells have been seeded onto biocompatible scaffolds and transplanted into injured spinal cords to form new functional neural connections and circuits. Following this, the damaged spinal cord is ultimately repaired and reconstructed [11,12].

Cells can provide new neurons to form new neural circuits and also secrete cytokines promoting the regeneration of the spinal cord axon [7,13]. To date, various cell seeding sources have been used for the treatment of SCI, including dendritic cells (DCs) [14], olfactory ensheathing cells (OECs) [15], schwann cells (SCs) [4]and neural stem cells (NSCs) [7,16] to name a few. Among these, NSCs are the most widely used for SCI with broad development prospects. Many studies on NSC transplantation have shown that, besides supplementing the loss of neurons and glial cells, NSCs may also play other roles during the promoting of the formation of new myelin [7,16]. Currently, various techniques are reported for obtaining NSCs, such as differentiation from embryonic stem cells (ESCs) [17] or induced pluripotent stem cells (iPSCs) and direct isolation from embryonic or adult mammal central nervous systems (CNS). However, these approaches are non-viable owing to ethical issues or potential tumorigenic risks. In our study, we induced mouse embryonic fibroblasts (MEFs) by directly reprogramming them into NSCs. According to extensive previous stuies, this method for obtaining NSCs not only avoids ethical issues and tumorigenic risks, but is also more simple and efficient compared with other techniques [18,19,20].

Another important aspect of tissue engineering is choosing an appropriate scaffold for seeding cells into for their adherence and growth. A biocompatible and biodegradable scaffold is key for tissue engineering to treat SCI [9,12,21]. Poly (lactic-co-glycolic acid) (PLGA) is one of the few biomaterials approved by the United States Food and Drug Administration for experimental and clinical application. It has been widely used as an artificial catheter, drug control-released carrier and tissue engineering scaffold material [22,23]. PLGA is one of the top biodegradable synthetic polymers used for tissue engineering owing to the ease of controlling its mechanical properties and biodegradation[22,24]. At present, PLGA-based nanofibers have made remarkable contributions in nerve regeneration [25]. Electrospinning has played a very important role in the construction of nanostructured materials over the past decade. Electrospun fibers made of natural polymers typically exhibit inferior mechanical properties. The superior pore features of electrospun fibers, such as fully interconnected pore structures, high bulk porosity and a wide open surface, facilitate water and nutrient transport desirable for cell growth [26,27]. In addition, owing to highly hydrophilic characteristics, low toxicity and immunogenicity, poly (ethylene glycol) (PEG) modification on PLGA scaffolds is an attractive tool for optimizing their biomedical applications [28,29]. In our study, we aimed to synthesize biofunctionalized electrospun PLGA-PEG nanofiber scaffolds and test their compatibility for the proliferation and differentiation of iNSCs *in vitro*. Furthermore, we transplanted the cell-seeded scaffolds into a SCI rat model to observe the growth and differentiation of the cells, and their effect on recovery of neural function.

## Materials and Methods

### Ethics Statement and Animals

All experimental protocols and animal handling procedures were approved by the Animal Care and Use Committee of Sun Yat-sen University (Approval Number: SYXK2012-0083), and were consistent with the National Institutes of Health Guide for the Care and Use of Laboratory Animals. Pregnant CD-1 mice were obtained from Beijing Vital River Experimental Animal Technology Co., Ltd. SD rats were supplied by Shanghai Slaccas laboratory animal co., Ltd.

### Preparation of low-attachment dishes

Dulbecco’s Modified Eagle Medium (DMEM) high glucose powder (13.4g, GIBCO) and sodium bicarbonate (3.7g) were dissolved in 500ml double diluted H_2_O (ddH_2_O) for preparation of 2×DMEM. 2×DMEM was sterilized through a 0.22μm filter. For preparation of 1% agarose solution, agarose G-10 (BIOWEST) was dissolved in ddH_2_O and heated until boiling in a microwave. Pre-warmed 2×DMEM medium and 1% agarose solution were mixed equally and added to a cell culture dish. The mixture of agarose and DMEM formed a 5mm thick layer of 0.5% gel when the solution cooled down.

### Preparation of MEFs

MEFs were isolated from E13.5–14.5 day CD-1 mouse embryos. Embryos were washed in phosphate buffered saline (PBS) three times. Embryo heads, limbs and spinal cords were carefully removed. The remaining embryos were cut into 1mm^3^ pieces. These pieces were then incubated in 0.25% trypsin with 1mM EDTA solution for 10 min. An equal amount of DMEM (GIBCO) with 10% fetal bovine serum (FBS) was added after incubation. Trypsinized tissues were left for 10 min at room temperature and then the supernatant was transferred into a new collection tube. Cells were harvested by centrifugation at 300g for 10 min and resuspended in DMEM with 10% FBS. MEFs were cultured at 37°C with 5% CO_2_. Passage 2‒3 (P2-3) MEFs were used for reprogramming.

### Preparation of WT-NSCs

WT-NSCs were isolated from E13.5–14.5 day CD-1 mouse embryos. Embryos were washed in phosphate buffered saline (PBS) for three times. Brains were separated and coronal blocks between rhinal fissure and hippocampus were isolated. The blocks was laid in a new dish and two parasagittal cuts were made by razor blade just lateral to the lateral ventricles. A horizontal cut was made to remove the tissue above the corpus callosum. This procedure leaves a small, rectangular chunk of tissue surrounding the lateral ventricles containing a high density of NSCs. Then, the tissue were washed in PBS for three times before cut into 1mm3 pieces. These pieces were then incubated in 0.25% trypsin with 1mM EDTA solution for 5 min at 37°C. An equal amount of DMEM (GIBCO) with 10% fetal bovine serum (FBS) was added to neutralize trypsin. Trypsinized tissues were left for 10 min at room temperature and then the supernatant was transferred into a new collection tube. Cells were harvested by centrifugation at 300g for 10 min and resuspended in DMEM/F12 supplement with B27 and N2 (Invitrogen) supplements, 20 ng/ml basic fibroblast growth factor (bFGF; PeproTech) and 20 ng/ml epidermal growth factor (EGF; PeproTech). WT-NSCs were cultured at 37°C with 5% CO2. Passage 5–8 WT-NSCs were used for further experiment.

### Sox2-retrovirus and GFP- retrovirus production and infection

The pMXs-Sox2 and pMXs-GFP plasmids were gifts from the Center for Stem Cell Biology and Tissue Engineering of Sun Yat-sen University. The Platinum E retroviral packaging cell line was kindly provided by the Cell-Gene Therapy Translational Medicine Research Center, the Third Affiliated Hospital of Sun Yat-sen University. Retroviruses were generated according to the recommendation of the manufacturer. P2-3 MEFs were seeded on a new dish one day before infection. MEFs were then transduced with Sox2 retrovirus for 24 h and cultured in MEF medium (DMEM+10%FBS). After infection (48 h), MEFs were collected and cultured in induction medium (DMEM supplemented with 15% FBS, 1mM L-glutamin, 1mM sodium pyruvate, 0.1mM nonessential amino acids (NEAA), 1× penicillin/streptomycin, 0.1mM β-mercaptoethanol (GIBCO) and 1000 units of recombinant leukemia inhibitory factor (LIF, Millipore) on prepared low-attachment dishes. The medium was changed every other day. After 7 days of reprogramming, cells were collected and seeded on gelatin-coated dishes. On reaching confluence, cells were collected and cultured on low-attachment dishes to form neurospheres. After three rounds of neurosphere formation, NSC-like cells were cultured and passaged in suspension culture. Then iNSCs were transduced with GFP retrovirus for 24 h and cultured in suspension culture for further experiment.

### Differentiation of iNSCs

2×10^4^ iNSCs was seeded onto gelatin-coated glass coverslips in 24-wells plates with NSCs medium (DMEM/F12 supplement with B27 and N2 (Invitrogen) supplements, 20 ng/ml basic fibroblast growth factor (bFGF; PeproTech) and 20 ng/ml epidermal growth factor (EGF; PeproTech). The culture medium was changed into NSC differentiation medium (DMEM/F12 supplement with 5% FBS, 1μmol/L RA, 1mM L-glutamin, 1mM sodium pyruvate, 0.1mM NEAA, 1× penicillin/streptomycin (GIBCO) 24h later and cultured for 7–14 days with a medium change of every other day.

### Immunocytochemistry

For immunofluorescence analyses, cells were fixed in 4% paraformaldehyde (Wuhan, Boster Biotech Co., Ltd., China) at 4°C for 10min. Then washed with PBS (Wuhan, Boster Biotech Co., Ltd., China) for three times, and incubated in blocking solution (6% fetal bovine serum(FBS), Life technologies) in PBS with 0.1% Triton X-100 (Sigma) for 1h at room temperature. The cells were then incubated with primary antibodies overnight at 4°C. Primary antibodies consisted of Nestin (ms IgG, 1:200; Abcam), MAP2 (rb IgG, 1:1000; Millipore), GFAP (rb IgG; 1:200; Santa Cruz), MBP (ms IgG; 1:200; Santa Cruz). Next morning, the cells were washed with PBS for three times and further incubated with secondary Alexa488- or Alexa555-labeled antibodies (1:400; Life technologie) for 60min at room temperature. Nuclei were detected by Hoechst 33342 (Sigma) staining. Micrographs were taken with a fluorescence microscope (Leica, Germany).

### RNA isolation and quantitative real-time PCR (qRT-PCR) analysis

Total RNA was isolated from cultured cells using Trizol (Invitrogen). One microgram of total RNA per sample was reverse transcribed using the Reverse transcription Kit (Takara) and the cDNA was diluted with 80μL of water. The diluted cDNA was used for quantitative PCR with MaximaTM SYBR Green/ROX qPCR Master Mix (Thermo). All qPCR reactions were done in triplicate, and all expression data were normalized to GAPDH expression. All primer sequences are listed in Table 1. qRT-PCR was performed using a Stratagene Mx3000P Realtime PCR system (Agilent).Gene expression relative to glyceraldehyde-3-phosphate dehydrogenase (GAPDH) was calculated using the 2-ΔΔCt method. 2-ΔΔCt>2 or <1/2 wasconsidered statistically significant.

**Table 1:** Primers used for QRT-PCR analyses.

GENE	Accession Numbers	Forward primer	Reverse primer
Blbp	S69799.1	CGCAACCTGGAAGCTGACA	GCCCAGAGCTTTCATGTACTCA
Ascl1	NM_008553.4	TCGTCCTCTCCGGAACTGAT	TAGCCGAAGCCGCTGAAGT
Zfp42	NM_009556	CCGGGATGAAAGTGAGATTAGC	TCACCTCGTATGATGCACTC
GAPDH	NM_008084	AGGTCGGTGTGAACGGATTTG	GGGGTCGTTGATGGCAACA
Nestin	NM_016701	GCAGAGTCCTGTATGTAGCCAC	AGAGTCAGATCGCTCAGATCC
Pax6	NM_013627	GTTGTGTGAGTAAAATTCTGGGC	GAGTCGCCACTCTTGGCTTA
Nanog	NM_028016	CACAGTTTGCCTAGTTCTGAGG	GCAAGAATAGTTCTCGGGATGAA

### Patch clamp analysis

iNSC-derived neurons were tested on a microscopic workbench with a patch clamp. Neurons were immersed in extracellular fluid containing 95% O_2_ and 5% CO_2_ during the whole process. Cells were visualized using an OLYMPUS patch clamp microscope. Medium-sized neurons with a bright cell margin and smooth surface were selected for patch clamp analysis. Neurons were inserted into the clamp and stimulated by an electric current and data were recorded using and AXON MultiClamp 700B amplifier and Igor 5.0 software.

### Preparation of electrospun PLGA and PLGA-PEG scaffolds

PLGA (75:25) and PEG (molecular weight 4000) were dissolved in hexafluoroisopropanol according to the proportion (15% and 1.5%), then placed in the 5mL plastic syringes with 0.4mm needles. The scaffolds were fabricated at an applied voltage of 15 kV with a voltage regulated DC power supply (DW-P203-1ACFD, Tianjin Dongwen High Voltage Power Supply Plant, China), and at a feeding rate of 1.0 mL/h. The distance between the collector and the needle was 12cm. Electrospun PLGA-PEG nanofibrous scaffolds were collected from receiving screen surface. Same method was used to produce electrospun PLGA nanofibrous scaffolds.

### Cell viability, adhesion and proliferation on electrospun scaffolds

Cell viability on PLGA-PEG scaffolds was evaluated by Cell Counting Kit-8 (Nanjing, KeyGEN Biotech Co., Ltd., China) according to the manufacturer’s procedure. Briefly, after cultured for 2 days, 100μl CCK-8 solution was added to each well (n = 3) and OD of the solution was measured at 450 nm (Elx800, Biotek, USA) after incubated for 2 h at 37°C.

iNSCs (1×10^4^ per well) were seeded onto PLGA-PEG scaffolds in 96-well plates. After incubation for 3, 6 and 9 hours at 37°C, scaffolds were washed in PBS for three times to remove the cells that did not adhere to the scaffolds. The remaining cells were collected with 0.25% trypsin with EDTA and counted under inverted optical microscope (NIKON TS100, Japan).

Cell proliferation in scaffolds was evaluated by Cell Counting Kit-8 (Nanjing, KeyGEN Biotech Co., Ltd., China) 1, 2, 3, 5, 7, and 9 days after seeding. Scaffolds were incubated with CCK-8 solution for 2 h at 37°C and OD of the solution was measured at 450 nm with the 96-well plates (Elx800, Biotek, USA). The proliferation curve of iNSCs on scaffolds was drawn.

### Scanning electron microscopy (SEM) morphological examination and nuclei staining

Scaffolds were fixed in 2.5% glutaraldehyde overnight at 4°C, and washed with PBS for three times, then dehydrated with a series of graded ethanol and freeze dried for 2 days. The dried samples were covered with gold using sputter coating (IB5 ion coater, EIKO, Japan). And the cell morphologies were observed with SEM (Quanta 200, FEI, USA).

### Preparation of 3D PLGA-PEG scaffolds for cell culture

**Fig 1:**
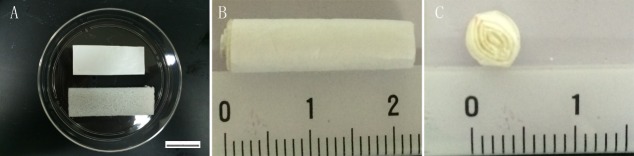
Preparation of 3D PLGA-PEG scaffolds. GS and PLGA-PEG film were used to assemble 3D PLGA-PEG scaffolds (A). PLGA-PEG film and GS were rolled together into a cylinder of 4mm diameter (B). The cylinder was cut transversely into 2 mm long cylinders (C).

Gelatin sponge (GS) and PLGA-PEG film were used to assemble the 3D scaffolds (Fig 1A). GS was firmly oppressed to make it thinner. The oppressed GS was placed above the PLGA and PLGA-PEG scaffolds. Scaffolds and GS were then rolled together into a cylinder, 4mm in diameter(Fig 1B). The cylinder was cut transversely into 2mm long cylinders and sterilized in 75% ethanol for 30 min, washed with PBS three times and stored in a dry and sterile environment before use(Fig 1C). The 3D scaffolds were soaked in culture medium for 10 min before use. Culture medium (20‒30 μl) containing 1×10^6^ cells was then injected into each 3D scaffold for cell-seeding. Scaffolds implanted with iNSCs were incubated at 37°C for 30 min in a 24-well plate before 500μL culture medium was added to each well. The medium was changed every other day.

### Cell distribution and survival within 3D scaffolds

1×10^6^ iNSCs were seeded on each 3D scaffold. After cultured for 7 days, cell distribution and survival within 3D scaffolds were evaluated after Hoechst33342 staining and calcein-AM/PI staining respectively. 10 μg/ml Hoechst33342 (Sigma), 2μmol/L calcein-AM and 4μmol/L PIwas added for nuclei staining, live cell staining and dead cell staining respectively. After incubation, scaffolds were washed with PBS for three times. Then scaffolds were fixed with 4% paraformaldehyde for 10 min before frozen section was performed. Peripheral and central parts of the scaffolds were chosen to evaluate the distribution and survival within 3D scaffolds. Sections were examined with fluorescence microscope (Leica, Germany).

### Spinal cord transection and transplantation

**Fig 2:**
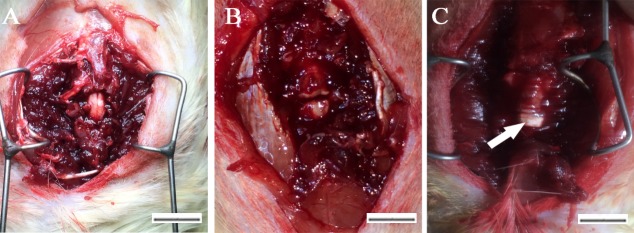
Surgical procedures of SCI. Spinal cord was transected after laminectomy at T10-11 level (A). A 2-mm spinal cord segment completely removed at T10 spinal cord level (B). Then, the 3D scaffolds of 2.0 mm in length with iNSCs were transplanted to the gap (C).

Thirty adult female SD rats (220g-250g, supplied by Slaccas laboratory animal co., LTD, Shanghai) were divided into three groups: PLGA group (n = 10), PLGA-PEG group (n = 10), and SCI group (n = 10). The rats were anesthetized with 10% chloral hydrate (0.3ml/100g) before surgery. The spinal cord was transected after laminectomy at T10-11 level (Fig 2A). A 2mm spinal cord segment completely removed at T10 spinal cord level (Fig 2B). Then, the 3D scaffolds of 2.0 mm in length with iNSCs were transplanted to the gap between the rostral and caudal stumps in PLGA and PLGA-PEG group (Fig 2C). As control, nothing was transplanted in SCI group.

For postoperative care, the bladder was emptied manually twice a day for 2 months. And all rats received intramuscular injection of penicillin (50,000 U/kg/day) for 3 days in order to prevent infection. Cyclosporine (10mg/kg/day) was used for the suppression of the immune rejection.

### Tissue processing

1 week and 2 months after operation, rats were sacrificed for tissue processing. All rats were anesthetized with 10% chloral hydrate before perfused transcardially with saline containing 0.002% heparin and 4% paraformaldehyde in 0.01M PBS (pH 7.4). The spinal cord were dissected and postfixed in 4% paraformaldehyde at 4°C for 24h, and then placed in 30% phosphate buffered sucrose at 4°C for 48h. Last, the spine cords were embedded in the optimum cutting temperature (OCT) compound (Sakura) and frozen in -80°C refrigerator before frozen sections. All spinal cord slices were cut at 10μm thickness.

### Cavity Assessment

Spinal cord sections of each group were stained with Hematoxylin and eosin (H&E) for cavity assessment. One in every five of the whole series of sections from each animal was selected for cavity areas analyses. The cavity areas of the spinal cord were analyzed by Image-Pro Plus software (Media Cybernet-ics, Silver Spring, MD). Cavities within 3 mm rostral or caudal to the graft site were measured, and those with a diameter less than 50μm were excluded. The total areas of all the cavities in every section were averaged for every experimental case. Measurement of cavity area was conducted blindly.

### Behavior Testing

Basso, Beattie, and Bresnahan (BBB) locomotor scale were used for the assessment of hindlimb locomotor recovery after SCI for all animals. Score ranges from 0 (no hindlimb movement) to 21 (normal hindlimb move). All observers were blinded to the operation procedure of the rats. And the test was performed one day post-operation and once every week up to the eighth week after operation.

### Statistical analysis

Data were represented as means±SDs. All statistical analyses were performed using the statistical software SPSS13.0. The data were analyzed using a student t-test when two group of data were compared, and one-way analysis of variance (ANOVA) was used when three group of data were compared. The significant level was set at 0.05.

## Results

### Generation of iNSCs form MEFs through 3D sphere culture

**Fig 3:**
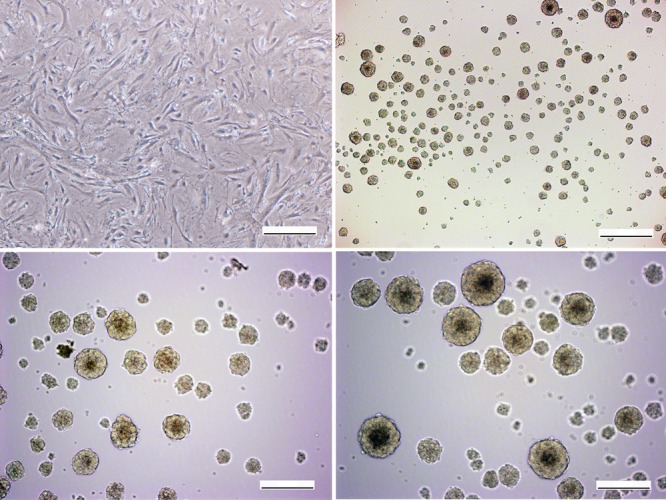
Generation of iNSCs. MEFs in monolayer culture for 7 days, no significant morphological change was observed (A). MEFs in low-attachment culture for 7 days, neurosphere-like clones were formed (B). iNSCs morphology was maintained over prolonged passaging at P8(C) and P20 (D) iNSC were similar to that of wt-NSCs. Scale bars = 200μm (A, C, D), 400μm (B).

MEFs were cultured in monolayer before induction (Fig 3A). Sox2-infected MEFs were then cultured in induction medium on low-attachment dishes and spheres were formed within 24 h. On day 7 of induction, the spheres highly resembled neurospheres (Fig 3B). These NSC-like spheres were collected for further passage and purification. Through several passages, iNSCs still possessed self-renewing ability and neurosphere morphology (Fig 3C and 3D).

### iNSCs gene expression signature

**Fig 4:**
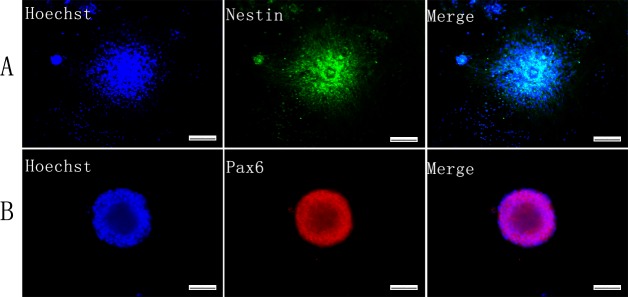
Identification of iNSCs by immunofluorescence. NSC markers were assessed by immunofluorescence, including Nestin (A) and Pax6 (B). Scale bars = 100μm (A, B).

**Fig 5:**
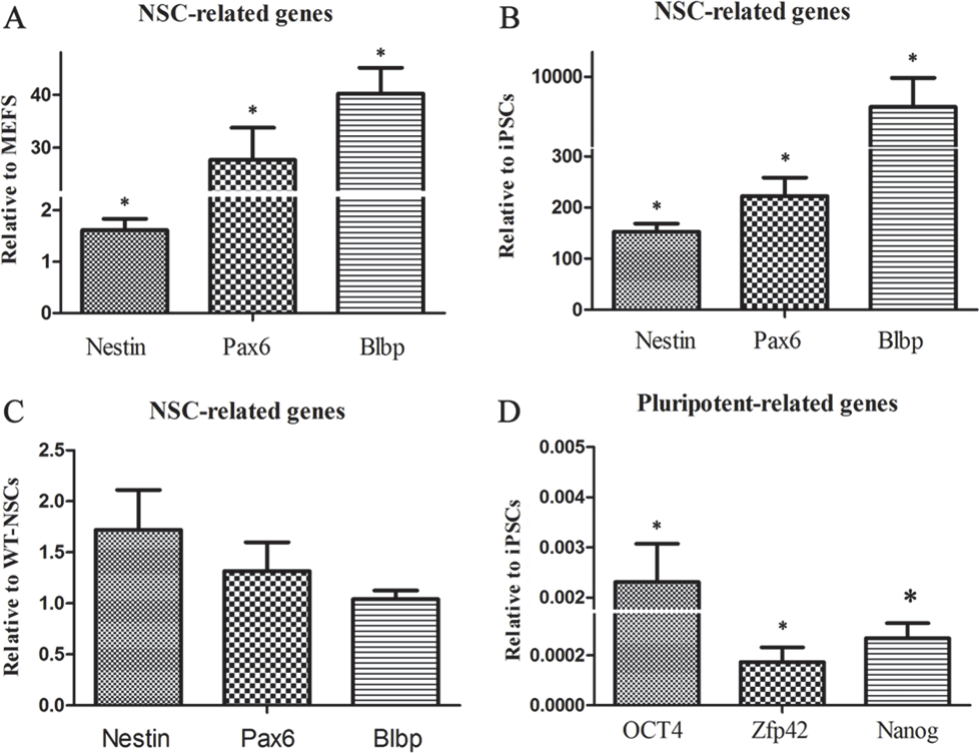
QRT-PCR analysis of iNSCs. QRT-PCR revealed that expression of typical NSC-related genes dramatically increased on the 7th day of induction compared to MEFs (A) and iPSCs (B) with negligible expression of pluripotency-related genes (D). Furthermore, iNSCs were similar to wt-NSCs in terms of expression of NSC-specific genes (C).

The reprogrammed NSC-like cells expressed NSC makers, including nestin and Pax6 (Fig 4A and 4B). Quantitative real-time polymerase chain reaction (QRT-PCR) analysis revealed that expression of NSC-related genes increased dramatically on the 7th day of reprogramming compared to monolayer MEFs (Fig 5A) and iPSCs (Fig 5B). Furthermore, expression of nestin, Pax6 and Blbp were similar to wt-NSCs (Fig 5C). However, they did not express pluripotency-related genes, such as Oct4, Nanog and Zfp42 (Fig 5D).

### Differentiation of iNSCs *in vitro*

**Fig 6:**
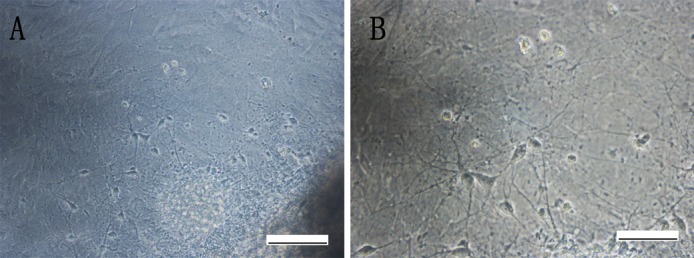
Differentiation of iNSCs in vitro under microscopic observation. Differentiated cells gradually migrated from neurospheres two days after adherence (A). Seven days later, neurites and differentiated cells were observed around the adherent iNSCs (B and C). Scale bars = 200μm (A), 100μm (B).

**Fig 7:**
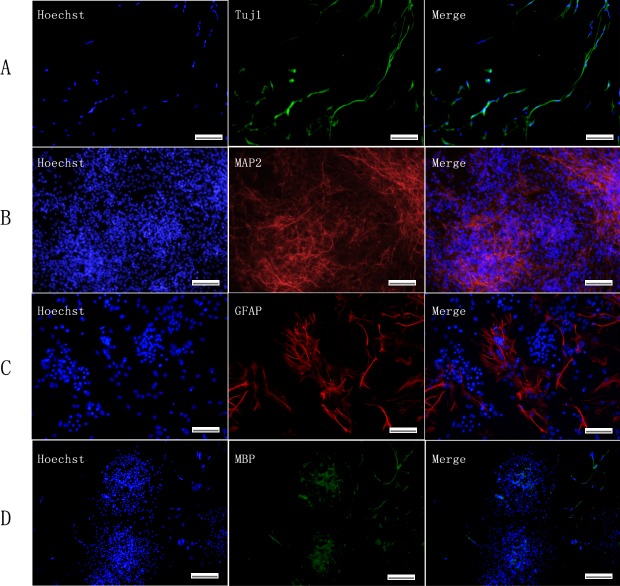
Immunofluorescence of differentiated iNSCs. Neurons, oligodendrocytes, and astrocytes were assessed by immunofluorescence for Tuj1 (A), MAP2 (B), GFAP (C), and MBP (D). Scale bars = 100μm (A, C), 200μm (B, D).

Next, we evaluated the differentiation potential of iNSCs into neurons, astrocytes and oligodendrocytes. When iNSCs were cultured onto gelatin-coated dished in the absence of epidermal growth factor (EGF) and fibroblast growth factor (FGF), but in the presence of FBS and retinoic acid, they exhibited neural lineage cell-like morphology on the 7th day (Fig 6A and 6B). Immunofluorescence staining also revealed Tuj1-positive neurons (Fig 7A). After further culture for 7 days, MAP2-positive mature neurons were also observed (Fig 7B). Furthermore, iNSCs developed into GFAP-positive astrocytes and MBP-positive oligodendrocytes (Fig 7C and 7D). At the 14^th^ day of differentiation, the percentage of differentiated neurons, astrocytes and oligodendrocytes was quantified. In the iNSCs group, the differentiation percentage of neurons, astrocytes and oligodendrocytes were 42.36±4.39%, 37.92±4.88% and 8.88±3.91% respentively (data not shown). In the WT-NSC group, the differentiation percentage of neurons, astrocytes and oligodendrocytes were 39.44±5.41%, 36.20±8.78% and 9.90±5.83% respentively (data not shown). In conclusion, iNSCs possessed multiple differentiation potential into neurons, astrocytes and oligodendrocytes.

**Fig 8:**
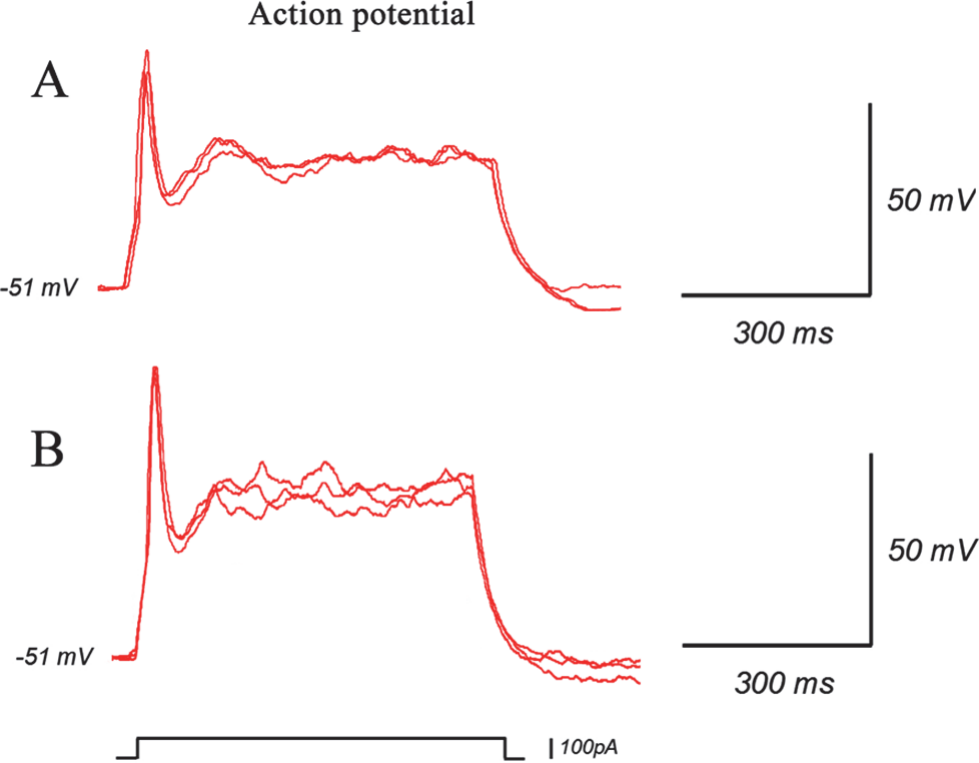
Electrophysiological function of differentiated neurons. The neurons differentiated from iNSCs exhibited functional membrane properties. Whole-cell patch-clamp recordings showed action potentials of iNSCs-derived neurons (B) which is similar to wt-NSCs (A).

In addition, the resulting neurons exhibited functional membrane properties. Whole-cell patch-clamp recordings were performed after 3 weeks of differentiation and 65.2% of the cells we detected showed action potentials (Fig 8), which was a similar level to wt-NSCs.

### iNSC survival, adhesion and process extension on PLGA-PEG nanofibers scaffolds

**Fig 9:**
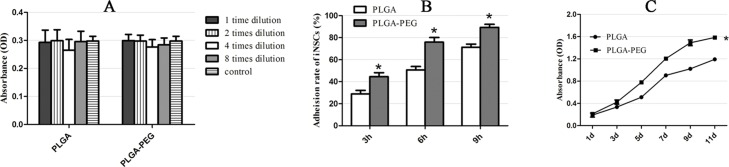
iNSCs survival, adhesion and process extension on electrospun nanofibers. Cell viabilities of iNSCs in both PLGA and PLGA-PEG group were no significant differences than blank (A). Cells adhesion rates on PLGA-PEG scaffolds were significantly higher than those of PLGA scaffolds at each time point (P<0.05) (B). And for the detection of cell proliferation, the numbers of iNSCs in PLGA-PEG group were significantly higher than that of PLGA group (P<0.05) (C).

iNSC cell viability on the seventh day in both the PLGA and PLGA-PEG group were not significantly different compared with the blank (Fig 9A). Cell adhesion rates on PLGA-PEG scaffolds were significantly higher than those on PLGA scaffolds at each time point (P<0.05) (Fig 9B). With respect to cell proliferation, cell number increased steadily from day 1 to 9 of incubation. Furthermore, numbers of iNSCs in the PLGA-PEG group were significantly higher than in the PLGA group (P<0.05) (Fig 9C).

**Fig 10:**
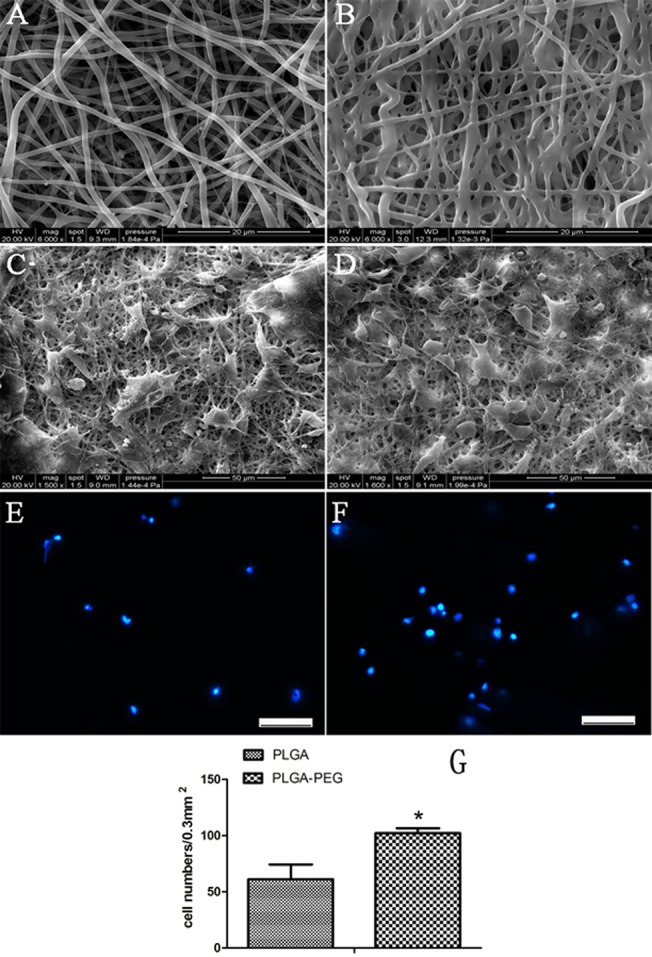
SEM morphological examination and nuclei staining. PLGA (A) and PLGA-PEG (B) nanobifers were observed by SEM. The results indicate that iNSCs differentiated into neural cells and neuritis grew better on PLGA-PEG electrospun nanofibers (D) compared to PLGA nanofibers (C). Hoechst staining shows normal morphology and quality of nuclei, no obvious cellular apoptosis or necrosis in PLGA-PEG groups (F). Besides, both of SEM and nuclei staining showed the cell population grew on PLGA-PEG scaffold was larger than that of PLGA scaffold (P<0.05 Student t-test, n = 5) (G). Scale bars = 50μm (E, F).

The morphometric SEM results indicated that iNSCs differentiated into neural cells, which grew well on both kinds of electrospun nanofibers (Fig 10C and 10D). Hoechst staining showed normal morphology and quality of nuclei, with no obvious cellular apoptosis or necrosis in both groups on the seventh day of co-culture (Fig 10E and 10F). Furthermore, nuclei staining showed the cell population on the PLGA-PEG scaffold proliferated to a greater extent than on the PLGA scaffold (P<0.05) (Fig 10G).

### Even distribution of iNSCs within 3D PLGA-PEG scaffolds

**Fig 11:**
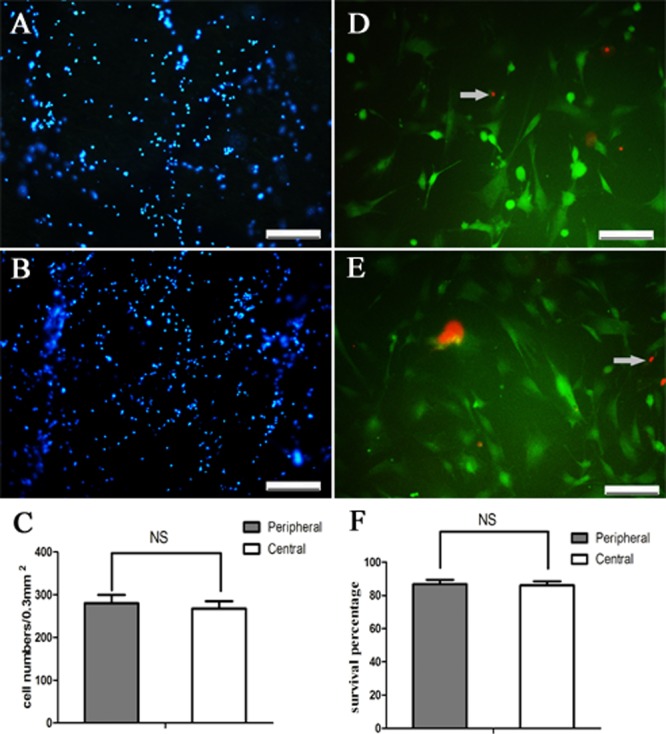
Distribution of iNSCs with the 3D PLGA-PEG scaffolds. On the seventh day, iNSCs that are stained with Hochest33342 were distributed evenly in peripheral (A) and central part (B). Furthermore, survival percentage of iNSCs on the seventh day in peripheral (D) and central part (E) showed no significant difference. NS indicate no significant difference between peripheral and central part (C, n = 5). Scale bars = 200μm (E, F).

Hoechst33342 staining of sections was performed to determine the distribution of iNSCs within the 3D scaffolds. Central and peripheral scaffold sections were examined and there was no significant difference in cell number between these areas (cell numbers per counting area (0.5×0.6 mm2): 280.2±43.23 vs. 267.6±38.53/counting area, p>0.05, Student t-test, n = 5). The above results showed that iNSCs were distributed evenly within the 3D scaffolds (Fig 11).

### Survival and differentiation of iNSCs within the PLGA-PEG scaffolds *in vivo*

**Fig 12:**
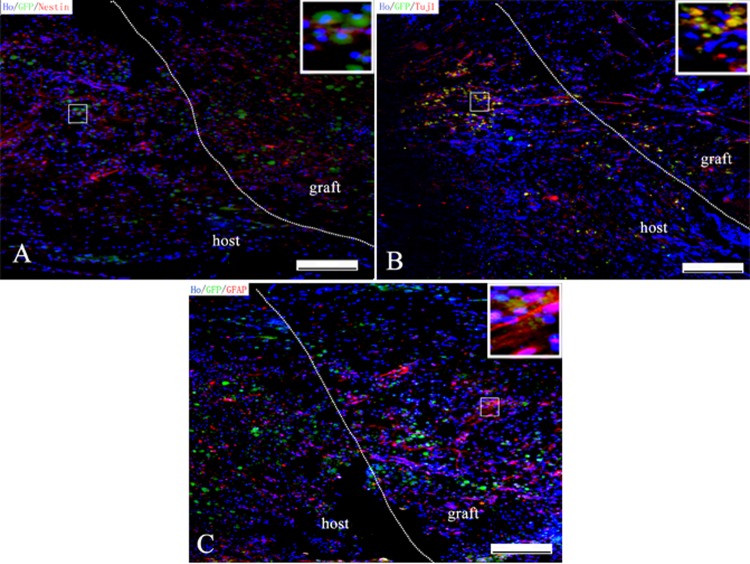
Immunostaining showing survival and differentiation of iNSC in the graft. Two week after transplantation, Nestin-positive iNSCs (A), Tuj1-positive neurons (B) and GFAP-positive glial cells (C) were observed in graft site and surrounding spinal cord in PLGA-PEG group. Scale bars = 200μm (A-C).

**Fig 13:**
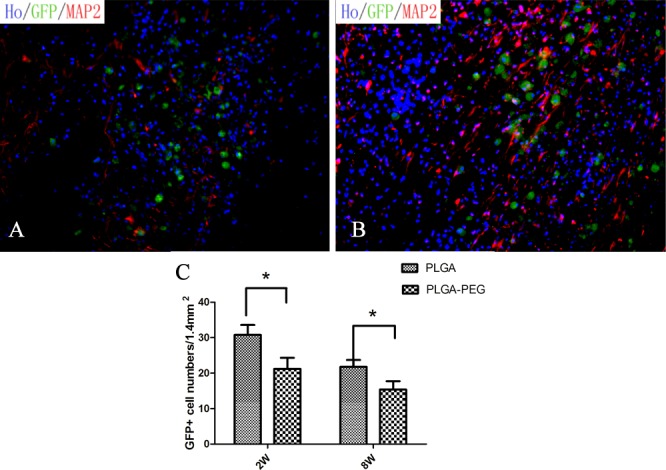
The long-term survival of transplanted cells in vivo. 8 weeks after transplantation, we can still see abundant MAP2/GFP-positive cells in PLGA-PEG group (B). However, only a small amount of GFP-positive cells was observed in PLGA group (A). Quantitative analysis revealed the numbers of GFP+ cells in PLGA-PEG group were significantly greater than PLGA group in 2 weeks and 8 weeks post-transplantation (P<0.05, Student t-test, n = 5) (C). Scale bars = 100μm (E, F)

We performed immunostaining through the spinal cord lesion to assess the survival and differentiation of iNSCs i*n vivo*. Two weeks post-implantation, it was observed that the implanted scaffold almost filled the lesion cavity and integrated with the host tissue. Double immunostaining of nestin/green fluorescent protein (GFP), MAP2/GFP and GFAP/GFP indicated that implanted iNSCs survived and differentiated into neurons and glial cells in the surroundings of the lesion site (Fig 12). Furthermore, 2 months post-operation, we observed abundant MAP2/GFP-positive cells in the PLGA-PEG group (Fig 13). Notably, the number of GFP-positive cells in the PLGA-PEG group was significantly higher than that of the PLGA group 2 weeks post-operation (GFP+ cell numbers per counting area (0.5×0.6 mm^2^): 160.60±18.72 vs. 91.20±11.61/counting area, *p*<0.05, Student t-test, n = 5) and 8 weeks post-operation (67.60±8.62 vs. 35.40±3.85/counting area, *p<*0.05, Student t-test, n = 5).

### iNSC-seeded PLGA-PEG scaffolds reduced cavity area

**Fig 14:**
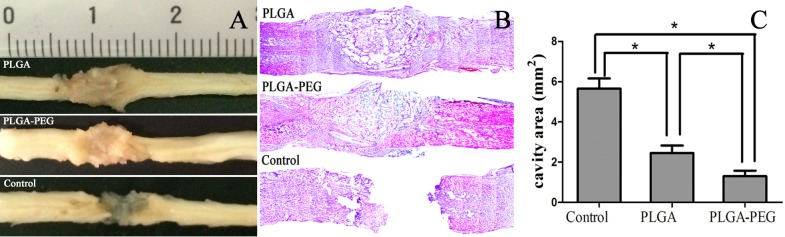
Gross morphology and cavity formation of spinal cord tissue dissected at 8 weeks post-operation. The PLGA and PLGA-PEG nanofibers both contributed to tissue structural integrity, whereas a large gap in the lesion site was still present in the control group (A). In both of PLGA and PLGA-PEG group, cavity areas were reduced compared with control group (B). The smallest cavity area was observed in PLGA-PEG group. However, larger cavities were observed in the PLGA group (P<0.05, Student t-test, n = 3) (C).

Eight weeks post-operation, grafts integrated better with the host tissue of the spinal cord by bridging rostral and caudal stumps in the PLGA-PEG group compared with the PLGA group (Fig 14A). Hematoxylin & eosin (H&E) staining was performed to evaluate the ability of the 3D scaffolds to reduce cavity area 8 weeks post-surgery. In both the PLGA and PLGA-PEG groups, cavity areas were reduced when compared with the control group (*p<*0.05). The smallest cavity area was observed in the PLGA-PEG group. However, larger cavities were observed in the PLGA group (*p<*0.05) (Fig 14B and 14C).

### Functional recovery post-SCI

**Fig 15:**
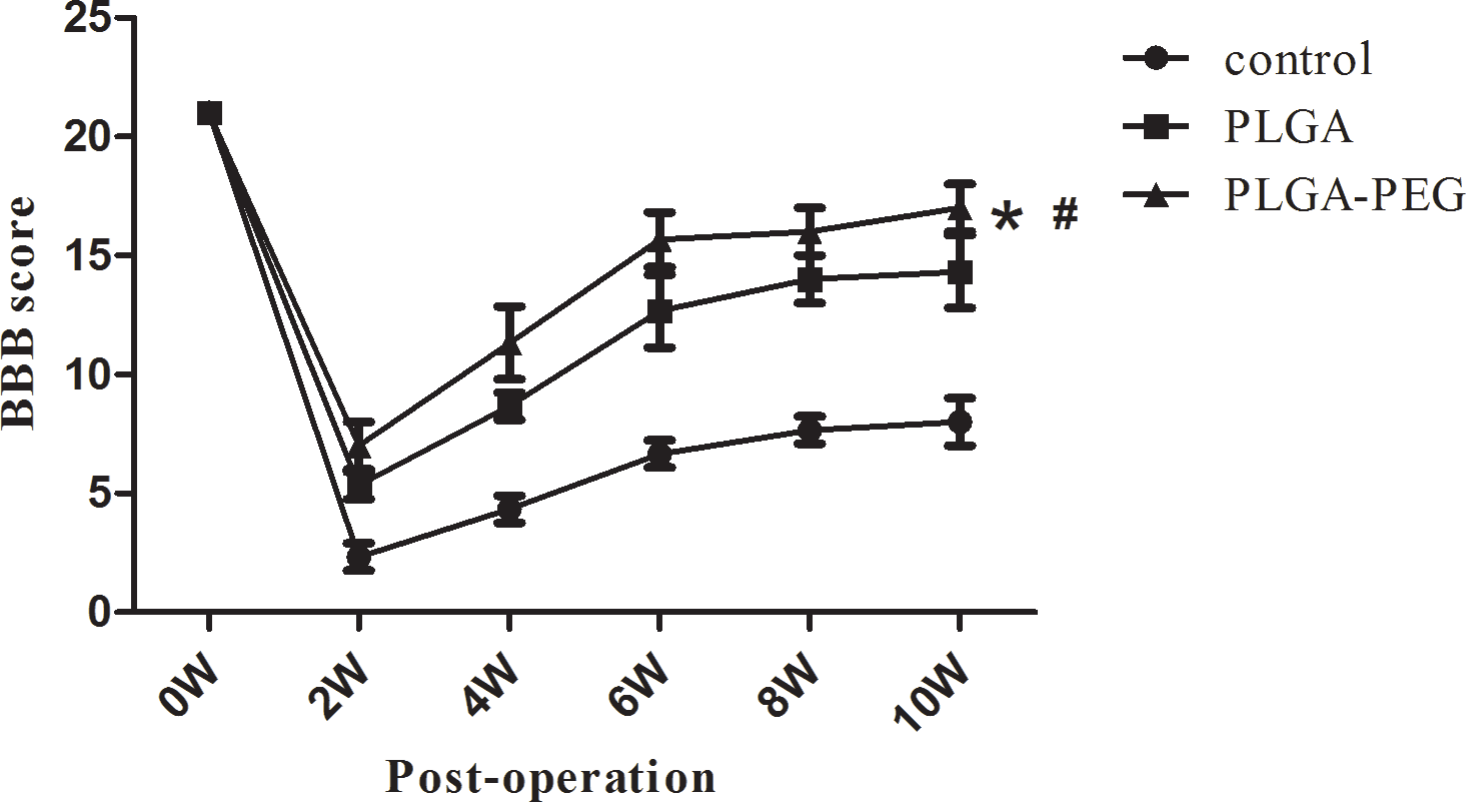
Behavioral outcome post-SCI. The functional recovery was improved in both of PLGA and PLGA-PEG group (*, P<0.05, one-way ANOVA, n = 10)). Notably, the functional recovery of the rats in PLGA-PEG group was better than PLGA group (#, P<0.05, one-way ANOVA, n = 10).

Behavioral analysis was performed using the Basso, Beattie, Bresnahan (BBB) Scale. Immediately after operation, BBB scores of all rats were nearly 0, indicating SCI model success. From 2 to 6 weeks post-operation, rapid functional recovery of rats was observed in the PLGA and PLGA-PEG groups. However, significant increase of the BBB score was observed in the control group. The results showed that functional recovery was improved in both the PLGA and PLGA-PEG groups. Notably, functional recovery of the rats in the PLGA-PEG group was better than in the PLGA group (Fig 15).

## Discussion

SCI is a severe disease resulting in functional damage and sensory loss [4]. Owing to primary mechanical damage and subsequent secondary injury after trauma, SCI often causes the death of local nerve cells and glial cells, as well as fracture and demyelination of nerve fibers [2,5,30]. It is therefore crucial to supplement neurons and glial cells for functional recovery after SCI. With the rapid development of stem cell research, stem cells can now be used for the treatment of SCI by supplying new cells [31]. Stem cells are well-known for their self-renewal ability and multilineage differentiation potential [13,32]. Theoretically, under optimum conditions, stem cells can differentiate into required cells to replace those that are lost. A variety of stem cells have been used in CNS repair, including ESCs [33], NSCs [7]and bone marrow mesenchymal stem cells (MSCs) [34]. A number of studies indicate that NSCs are a suitable choice for the treatment and replacement of injured spinal cord. NSCs can efficiently differentiate into neurons, astrocytes and oligodendrocytes. Furthermore, owing to their low immunogenicity profile, NSCs do not cause obvious graft rejection after transplantation [17,20,35]. Various methods are available to obtain NSCs; their direct isolation from the CNS is the most widely used method at present. However, obtaining CNS samples remains a challenge [36]. Besides CNS-derived NSCs, differentiation from ESCs and iPSCs have also been widely used in recent years. However, the ethical debate surrounding these cell types continues, and tumorigenicity is also a potential threat [18,20,32]. In our study, we induced Sox2-transfected MEFs through direct reprogramming into neural stem cells on low-attachment surfaces. On the 7th day of induction, expression of NSC-related genes increased dramatically compare to original cells, which was similar to wt-NSCs. Furthermore, the lack of pluripotent-related gene expression in the iNSCs suggested that direct conversion of fibroblasts into iNSCs did not undergo an intermediate stage into ESCs. Furthermore, iNSCs exhibited a similar morphology to wt-NSCs, and were able to differentiate into neurons and glial cells *in vitro*. In addition, after transplantation of iNSCs-seeded 3D scaffolds into rats, cells were observed to survive and differentiate into neurons and glial cells. Notably, iNSCs have no ethical controversy attached to them, and are less likely to cause teratomas. In conclusion, iNSCs are the perfect cell substitute for CNS cell therapy, when compared with ESCs and iPSCs-derived NSCs. This approach will further promote the application of NSCs in the treatment of SCI.

In recent years, the rapid development of tissue engineering has promoted the invention and improvement of tissue engineering in spinal cord regeneration [9,11,12,37]. The general procedure of tissue engineering is to co-culture functional cells and biodegradable scaffolds *in vitro*, and then implant the constructs into damaged organs, thus replacing injured tissues to restore the structure and function of the original organ [23,38]. In addition, to select a suitable cell source, choice of appropriate scaffold is crucial. An optimal scaffold should provide mechanical support and a suitable environment for cell adhesion and growth [39,40]. PLGA is a functional polymer organic compound. Because of its good biocompatibility, non-toxicity, good film-forming ability and biodegradability, PLGA is widely used in the pharmaceutical, medical engineering and modern industrial fields [22,23]. However, there are also some shortcomings of PLGA nanofibers, including hydrophilic deficiency and reduced cell adhesion and proliferation, which require further improvement. PEG, composed of repetitive oxygen vinyl, is commonly used as a scaffold because of its hydrophilic property. PEG has been shown to protect key axonal cytoskeleton proteins to promote repair in SCI [28]. Electrospinning is a technique that enables alignment of nanofibers, which is controlled by increasing the collector rotating speed [24,41]. Such alignment better fits the needs for nerve supporting structure development. In our study, we synthesized an electrospun PLGA-PEG biomaterial and tested its ability to promote adhesion, proliferation and neurite outgrowth of iNSCs *in vitro*. The results indicated that the PLGA-PEG nanofibers scaffold was superior for iNSC adhesion and proliferation compared with the PLGA scaffold. Furthermore, the PLGA-PEG scaffolds showed no obvious cell toxicity. SEM results indicated that iNSCs differentiated into neural cells and neurites and grew well on both kinds of scaffolds. Hoechst staining showed normal morphology and quality of nuclei, with no obvious cellular apoptosis or necrosis on the scaffolds. Both SEM and nuclei staining showed that cell growth on PLGA-PEG scaffolds was greater than that on PLGA scaffolds. Based on this, electrospun PLGA-PEG nanofibers were found to be better scaffolds than PLGA for iNSCs growth, adhesion and proliferation. Because the spinal cord is a cylindrical organ, a cylindrical scaffold is essential for its tissue-engineered reconstruction. GS has served as a common scaffold in tissue repair owing to its affinity for cells and histocompatibility. We therefore put electrospun PLGA-PEG nanofibers and GS together to construct a 2mm-long cylinder with 4mm diameter. This 3D scaffold is reported to restore the continued morphology of spinal cord and provide a good support structure, facilitating cell growth and communication. We next seeded iNCSs onto the 3D scaffolds to form tissue-engineered spinal cords. The iNSCs and 3D scaffolds were co-cultured for 7 days *in vitro* before being transplanted into a rat spinal cord transection injury model. Hoechst staining revealed that iNSCs were evenly distributed within the 3D PLGA-PEG scaffold. Two weeks post-operation, the implanted scaffolds almost filled the lesion cavity and integrated with the host tissue. Immunostaining indicated the implanted iNSCs could survive and differentiate into neurons and glial cells in the surroundings of the lesion site. Furthermore, 8 weeks post-operation, we could still see abundant MAP2/GFP-positive cells. This confirmed the long-term survival and multiple differentiation potential of iNSCs within our 3D scaffold *in vivo*. Notably, the number of GFP-positive cells in the PLGA-PEG group was significantly higher than that of the PLGA group 2 weeks post-operation, indicating that the PLGA-PEG scaffold was more conducive to cell survival *in vivo*. Furthermore, the BBB score revealed that functional recovery was improved in both the PLGA and PLGA-PEG groups compared with the control group. Notably, functional recovery of the rats in the PLGA-PEG group was better than in the PLGA group. In conclusion, the 3D electrospun PLGA-PEG scaffold is suitable for the construction of tissue-engineered spinal cord.

SCI is a very complicated disease involving multiple factors. In our study, we only carried out preliminary study on the cell-seeding source and scaffold materials. However, there remain many problems and difficulties that need to be investigated and overcome. To provide adequate cells, the induction efficiency of iNSCs needs to be improved significantly. Moreover, Sox2-retrovirus was used to trigger reprogramming, which could pose a safety risk. Therefore, an efficient non-viral induction method needs to be investigated.
